# The Prevalence and Epidemiology of *Salmonella* in Retail Raw Poultry Meat in China: A Systematic Review and Meta-Analysis

**DOI:** 10.3390/foods10112757

**Published:** 2021-11-10

**Authors:** Tianmei Sun, Yangtai Liu, Xiaojie Qin, Zafeiro Aspridou, Jiaming Zheng, Xiang Wang, Zhuosi Li, Qingli Dong

**Affiliations:** 1 School of Health Science and Engineering, University of Shanghai for Science and Technology, Shanghai 200093, China; usststm@163.com (T.S.); usstlyt@163.com (Y.L.); qxj19900709@163.com (X.Q.); usstzjm@163.com (J.Z.); xiang.wang@usst.edu.cn (X.W.); lizhuosi624@163.com (Z.L.); 2 Laboratory of Food Microbiology and Hygiene, Department of Food Science and Technology, School of Agriculture, Faculty of Agriculture, Forestry and Natural Environment, Aristotle University of Thessaloniki, 54124 Thessaloniki, Greece; zasprido@agro.auth.gr

**Keywords:** foodborne pathogen, salmonellosis, chicken, antibiotic resistance, microbial contamination, food safety

## Abstract

Foodborne disease caused by *Salmonella* is an important public health concern worldwide. Animal-based food, especially poultry meat, is the main source of human salmonellosis. The objective of this study was to evaluate the prevalence and epidemiology of *Salmonella* contamination in raw poultry meat commercialized in China. Following the principle of systematic review, 98 sets of prevalence data were extracted from 74 publications conducted in 21 Chinese provincial regions. The random-effect model was constructed for subgrouping analysis by meat category, preservation type, and geographical location. The prevalence levels differed from high to low among raw poultry meat, including chicken, 26.4% (95% CI: 22.4–30.8%); pigeon, 22.6% (95% CI: 18.2–27.8%); duck, 10.1% (95% CI: 5.3–18.2%); and other poultry meat, 15.4% (95% CI: 12.0–19.5%). Prevalence data on the preservation type revealed that chilled poultry meat might be more likely to experience cross-contamination than non-chilled poultry meat in China. The distribution map of *Salmonella* for raw poultry meat showed that a higher prevalence level was found in the Shaanxi, Henan, Sichuan, and Beijing regions. All subgroups possessed high amounts of heterogeneity (*I*^2^ > 75%). The scientific data regarding the differences in prevalence levels between meat category, preservation method, and geographical region sources might be useful to improve specific interventions to effectively control the incidence of *Salmonella* in poultry meat.

## 1. Introduction

*Salmonella*, one of the most important foodborne pathogens in the world, is frequently implicated in foodborne disease outbreaks. It is estimated that *Salmonella* is responsible for approximately 1.3 billion cases of salmonellosis worldwide each year [1]. China has a high incidence of salmonellosis [2]. It was found that approximately 70% to 80% of foodborne diseases are caused by *Salmonella* in China [3]. Epidemiological studies have suggested that foods of animal origin, especially poultry and poultry products, are common vehicles of *Salmonella* transmission to human beings [4,5,6].

The monitoring and tracking of *Salmonella* in poultry meat and the establishment of efficient surveillance systems are the basis for effective public health protection and food safety management. In Europe, a baseline survey was conducted to estimate the prevalence of *Salmonella* and *Campylobacter* on broiler carcasses in 2008 [7]. In the USA, the United States Department of Agriculture Food Safety Inspection Service (USDA/FSIS) has established a verification program to inspect raw poultry products for the presence of *Salmonella* and *Campylobacter* [8]. In China, numerous studies investigated retail chicken meat for *Salmonella* contamination, which showed that up to 50% of retail chicken samples were contaminated with *Salmonella* [9,10]. However, limited information is available concerning *Salmonella* contamination of other poultry meats, such as duck, goose, and pigeon. Furthermore, these previous studies only included samples from one or a few regions from the whole territory of China. Given the variations in data availability and quality observed in the European Union [11], it is expected that the prevalence and contamination level will be different among the various regions of China. As such, there is a lack of comprehensive data on *Salmonella* contamination in poultry meat at the retail level in the whole region of China.

Meta-analysis is concerned with the statistical summary of a large number of results from multiple individual studies on a specific research question [12]. With meta-analysis, it may be possible to explain the sources of heterogeneity and differences among the findings of the primary research [13]. At present, the amount of data generated by food safety research is growing increasingly. In the field of food safety, meta-analysis is a valuable tool to deal with a broad range of research interests, such as disease incidence, epidemiology and prevalence of microorganisms, effect of pre- and post-harvest interventions, consumer practices, etc. [14,15]. Thus, meta-analysis results are an important part of quantitative microbial risk assessments (QMRAs), as they can provide more accurate data for risk assessment models than estimates based on a single study or expert opinion.

According to the Food and Agriculture Organization (FAO) [16], China’s poultry production is second only to the USA, and its consumption is increasing steadily. Recent poultry-related systematic reviews and meta-analyses were conducted to estimate the prevalence of *Salmonella* in poultry samples from Europe and North America [5,17,18,19,20]. To our knowledge, there is a lack of meta-analysis studies to estimate the pooled prevalence of *Salmonella* in retail raw poultry meat in mainland China. The current study attempted to generate pooled prevalence data based on existing publications from China using the meta-analytical approach. The objective of this study was to quantify *Salmonella* prevalence in Chinese retail poultry meat, to analyze the differences in *Salmonella* prevalence among sub-categories, and to evaluate the levels of the heterogeneity of the published prevalence data.

## 2. Materials and Methods

### 2.1. Search Strategy and Selection Criteria

Two databases were systematically searched, including the Web of Science (WoS) database and the China National Knowledge Infrastructure (CNKI) database. The following search strategy was carried out for collecting potentially relevant publications from the WoS database: (“prevalence” OR “incidence” OR “occurrence” OR “quality” OR “contamination” OR “survey” OR “sampling” OR “character*” OR “quanti*” OR “epidemiol*” OR “isolate” OR “enumerate*”) AND “Salmonella” AND (“chicken” OR “broiler” OR “duck” OR “goose” OR “turkey” OR “poultry” OR “meat”) AND (“China” OR “Chinese”) AND (“1950:2019”). Another search format for the CNKI database was: “Salmonella” AND (“contamination” OR “monitoring” OR “checking out” OR “inspection” OR “detection” OR “isolation” OR “epidemiological”) AND (“chicken” OR “duck” OR “goose” OR “poultry” OR “meat”) AND (“1979:2019”); all the terms were used in Chinese. To avoid missing any additional data, we conducted a complementary literature search on the reference list of relevant publications.

The PRISMA statement (Preferred Reporting Items for Systematic Reviews and Meta-Analyses, http://www.prisma-statement.org/ accessed on 16 November 2020) was employed for reporting the screening process. After removing duplicate records, all the publications were checked against a set of exclusion criteria. A study was excluded if (1) it was published as a conference abstract or was not a research paper (review); (2) it was not relevant, such as studies focusing on the detection method, predictive modeling, or hurdle technology; (3) it was a duplicate report; (4) the poultry samples were imported products; (5) the meat category was not clearly indicated; (6) incomplete data on the prevalence and concentration of *Salmonella* on poultry were reported; (7) the samples were not limited to the retail stage; and (8) the sample size was lower than 50.

### 2.2. Data Extraction

Data for *Salmonella* prevalence on raw poultry were extracted from the studies identified through the systematic review of the literature independently by a single reviewer and validated by a second reviewer. The following data were extracted from each eligible study of records: author, publication year, survey year, meat category, preservation type, sampling location (i.e., provincial, region), detection method, sample size, positive sample number, identified *Salmonella* serovars, the antimicrobial resistance rate of *Salmonella* to each antibiotic. The poultry meat was further categorized into ‘Chicken’, ‘Duck’, ‘Pigeon’, ‘Goose’, and ‘Other’. The preservation type category was subdivided into ‘Ambient’, ‘Chilled’, ‘Frozen’, and ‘Unknown’.

### 2.3. Meta-Analysis and Statistical Analyses

The meta-analysis and forest plot generation of this review were performed using R language (Version 3.4.3, http://www.R-project.org/ accessed on 6 March 2021) with the ‘meta’ package. For further subgroup analysis, data were grouped by the meat category, preservation type, and sampling location. Due to the fact that the sampling methods and experimental methodologies of the primary studies were not identical, the description of the heterogeneity (or variability) is critical in a meta-analysis [19,21]. As stated by Gonzales-Barron [13], a fixed-effect model may be unsuitable for application in the meta-analysis of the variability of food research. Thus, all eligible information in our study was pooled and analyzed on the basis of a random-effects model [22]. Cochran’s Q test and *I*-squared index (*I*^2^) were used for evaluating heterogeneity among studies [23]. The statistical significance for heterogeneity using Cochran’s Q test was defined for *p* < 0.10, and the degree of heterogeneity using *I*^2^ was defined as low, moderate, and high when *I*^2^ values (as percentages) were around 25%, 50%, and 75%, respectively [21]. The statistical map was generated based on the Chinese standard geographical map (downloaded at http://bzdt.ch.mnr.gov.cn, accessed on 1 April 2021).

## 3. Results

### 3.1. Characteristic of Literature and Datasets

**Figure 1:**
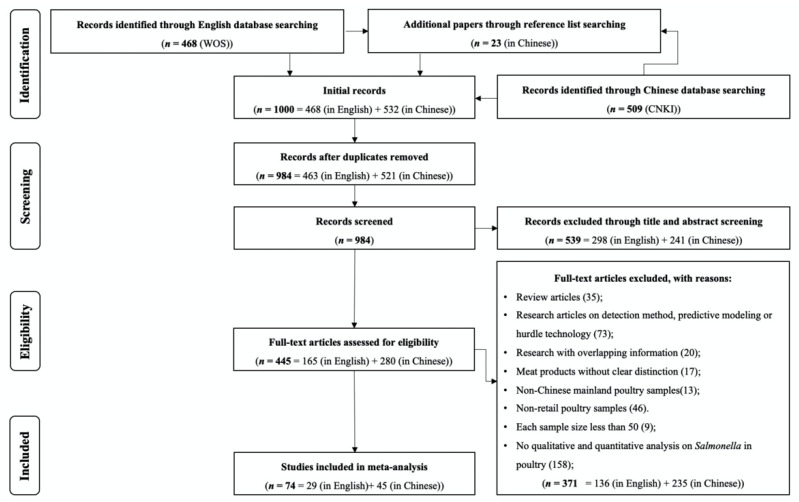
The flowchart of the literature searching and collecting.

The process for the selection of eligible articles is depicted in Figure 1. A total of 1000 publications were initially identified from the two selected databases. After removing duplicates and manual screening based on the specified criteria, 74 publications (29 in English and 45 in Chinese) of independent studies of *Salmonella* in retail poultry (before 2020) in China were finally included in our systematic review. Following full-text quality checking, a total of 98 sets of prevalence data of *Salmonella* in poultry meat were retrieved. The data encompassed a total of 21,824 samples (5837 positives) from 21 Chinese provinces, major municipalities, and autonomous regions. Due to the limitations of the included information, the origin of retail poultry meat is unknown (e.g., farm household or industry). Most samples belonged to the ‘Chicken’ category (*n* = 15,246), followed by the ‘Duck’ category (*n* = 794), ‘Pigeon’ category (*n* = 292), and ‘Other’ category (*n* = 5492). For qualitative or quantitative analysis of *Salmonella*, the pre-enrichment culture and identification process mainly referred to the Chinese national standard GB 4789.4 (versions 2003, 2008, 2010, and 2016) and a few studies (7 out of 74) deployed ISO 6579 or the Most Probable Number (MPN) method.

**Figure 2:**
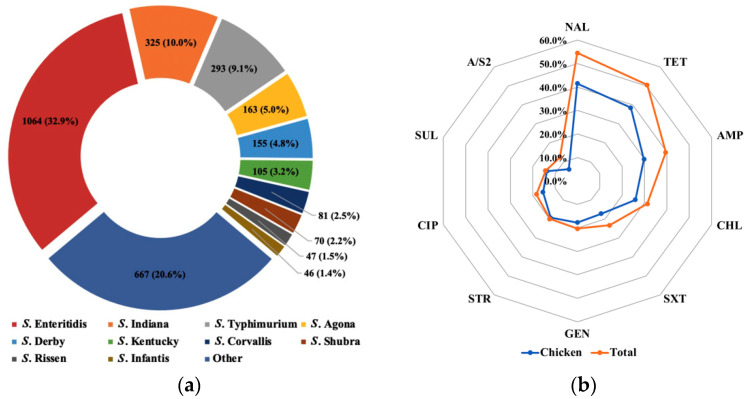
Serovar distribution (**a**) and antimicrobial resistance (**b**) of *Salmonella* strains isolated from Chinese retail raw poultry meat.

A total of 42 studies (20 in Chinese and 22 in English) reported the serotype analysis information of *Salmonella* isolates. As shown in Figure 2a, according to the serotyping of 3104 *Salmonella* isolates recovered from 13,119 poultry samples, the three most commonly isolated serovars were *S*. Enteritidis (32.9%), *S*. Indiana (10.0%), and *S*. Typhimurium (9.1%), followed by *S*. Agona (5.0%), *S*. Derby (4.8%), *S*. Kentucky (3.2%), *S*. Corvallis (2.5%), *S*. Shubra (2.2%), *S*. Rissen (1.5%), and *S*. Infantis (1.4%). All serovars were mostly isolated from chicken.

Antimicrobial susceptibility of *Salmonella* isolates was evaluated in a total of 27 studies (10 in Chinese and 17 in English). The antibiotic resistance rate was evaluated by dividing the number of resistant *Salmonella* isolates by the number of total *Salmonella* isolates (presented as percentage), when resistant *Salmonella* strains were present. Among the 2249 *Salmonella* isolates from 8920 poultry samples, the results for antimicrobial resistance rates of *Salmonella* are depicted in Figure 2b. The resistance most commonly detected was to nalidixic acid (54.6%), followed by tetracycline (50.6%), ampicillin (39.5%), chloramphenicol (31.4%), trimethoprim/sulfamethoxazole (23.3%), gentamicin (20.3%), streptomycin (20.1%), ciprofloxacin (18.3%), sulfisoxazole (14.2%), and ampicillin/sulbactam (12.4%). Chicken-derived isolates were the majority, and the levels of resistance of them to the above ten antibiotics were basically consistent with the total *Salmonella* isolates.

### 3.2. Salmonella Prevalence in Different Poultry Meat Product Types

The meta-analysis results on the prevalence and heterogeneity of *Salmonella* in poultry meat by poultry type are presented in Table 1. Overall, the pooled prevalence of *Salmonella* in raw poultry meat was 23.0% (95% CI: 19.8–26.8%), with heterogeneity (as indicated by the inverse variance index) as high as 97.0%. Among the different poultry meat categories, chicken presented the highest mean pooled prevalence (26.4%, 95% CI: 22.4–30.8%), followed by pigeon (22.6%, 95% CI: 18.2–27.8%) and duck (10.1%, 95% CI: 5.3–18.2%). Heterogeneity values were relatively low for the prevalence levels reported for pigeon (0%) and duck (87.9%), which may be due to the small number of related studies. In addition, due to the limited information in the included literature on whether the poultry samples were whole carcasses or anatomical pieces (legs, wings, etc.), it was impossible to ascertain the relationship between *Salmonella* prevalence and whole carcasses or anatomical pieces.

### 3.3. Salmonella Prevalence in Different Geographical Regions

**Figure 3:**
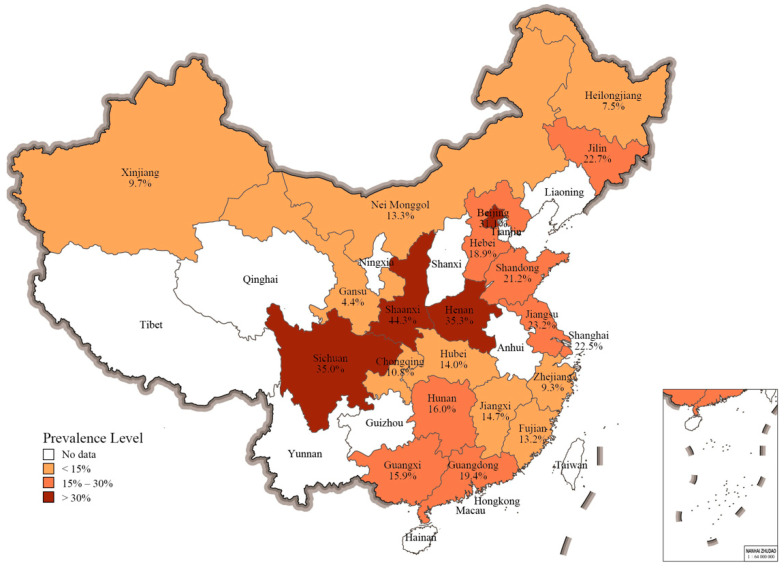
The pooled prevalence of *Salmonella* in raw poultry meat from 21 Chinses provincial regions based on the included reports.

Due to the inherent limitations of literature retrieval in meta-analyses, the prevalence data of *Salmonella* covered 21 provinces, major municipalities, and autonomous regions in China, occupying a land area of 6,558,251 km^2^ (approximately two-thirds of the total). The pooled prevalence estimates of *Salmonella* in poultry meat, to be presented as follows, cannot be generalized to other Chinese regions. The range of *Salmonella* prevalence levels found in raw poultry meat for those regions (indicating low (<15%), medium (≥15% and ≤30%), and high level (>30%) are shown in Figure 3. The highest prevalence level of *Salmonella* in raw poultry meat was reported in Shaanxi (44.3%, 95% CI: 29.9–59.7%), followed by Henan (35.3%, 95% CI: 21.2–52.5%), Sichuan (35.0%, 95% CI: 26.4–44.7%), and Beijing (31.1%, 95% CI:16.5–50.8%).

### 3.4. Salmonella Prevalence under Different Preservation Types

The results of the meta-analysis on the prevalence and heterogeneity of *Salmonella* by preservation type are shown in Table 2. The present study recorded 2825 ambient poultry meat samples, 2066 chilled poultry meat samples, and 2173 frozen poultry meat samples, and their pooled prevalence of *Salmonella* were 17.2% (95% CI: 6.6–37.8%), 42.1% (95% CI: 33.7–51.0%), and 25.3% (95% CI: 17.3–35.4%), respectively. Notably, *Salmonella* prevalence in chilled poultry meat was statistically higher than that of frozen poultry meat and ambient poultry meat. Among the included publications, the preservation method was unknown for more than half of the samples (14,760/21,824). In this fraction of the poultry meat samples, the pooled *Salmonella* prevalence was 21.3% (95% CI: 17.9–25.1%). A high heterogeneity was observed among each group.

## 4. Discussion

Microbiological foodborne hazards have attracted the attention of the food safety management system in China [24]. The Chinese Food Safety Law implemented in 2019 has legally clarified the roles and duties of the national food safety surveillance system for foodborne pathogens in foods [25]. In 2010, this national food safety surveillance system covered all 31 provinces, major municipalities, and autonomous regions in China, to support early detection, diagnosis, and management of foodborne pathogens [26]. Since then, a downward trend is apparent from the publicly available reports on the incidence of foodborne pathogens in foods [27]. However, reducing the incidence of foodborne diseases is a constant topic of concern for the Chinese government as well as the public.

This meta-analysis review demonstrated the widespread prevalence of *Salmonella* in retail poultry meat in China. The contaminated retail poultry may become an issue of concern because the products can be in direct contact and be used by consumers. Although raw meat generally receives a certain lethal treatment (e.g., conventional cooking, microwaving, etc.) before consumption, cross-contamination incidents and undercooking are still the greatest risks in consumers’ kitchens [28,29]. In the present study, the pooled prevalence of *Salmonella* in raw poultry meat in China was 23.0%, which is significantly higher than that reported in retail poultry from the European Union (7.1%) [5] and Africa (13.9%) [30]. Thus, raw poultry meat in retail may be an important source of human salmonellosis in China.

According to the prediction by the Organization for Economic Co-operation Development and the Food and Agricultural Organization (OECD-FAO) [31], poultry meat will continue to be the primary driver of meat production growth over the next ten years. Low production costs, a short production cycle, high feed conversion ratios, and low product prices have contributed to making poultry the meat of choice for both producers and consumers. Regarding the different poultry meat categories, chicken is the greatest concern as it bears the highest pooled prevalence of *Salmonella*. The high prevalence of *Salmonella* in raw chicken samples in our study suggests that chicken may be the main vehicle of transmission for *Salmonella* in China. In China’s meat consumption structure, chicken takes the largest proportion in poultry meat consumption and is on the rise, becoming the second-largest meat product after pork [32]. Similarly, in Chinese poultry farming operations, densities are generally higher for chickens, while they are considerably lower for ducks and geese (111.2, 27.4, and 6.7 thousand per km^2^ maximum, respectively) [33]. Thus, in response to potential public health pressures, more effective intervention strategies during processing should be implemented to control the quality and safety of chicken products.

In terms of geographical distribution, the occurrence of *Salmonella* in retail raw poultry meat is common in China. The pooled prevalence of *Salmonella* in poultry meat samples is the highest in Shaanxi, followed by Henan, Sichuan, and Beijing. There is no known scientific rationale for the observed geographical differences in the prevalence levels of *Salmonella*. From the spatial distribution of poultry animals in China, chickens are most ubiquitous, with high densities across much of eastern China, particularly the Yellow River Basin. Duck densities are highest in southeastern China and the Sichuan Basin [33]. Notably, farm practices can affect the prevalence of *Salmonella* in the final product [34]. Moreover, because the cold chain coverage of agricultural products in China is still much lower (20.0%) than that in developed countries (90.0%) [35], the supply of poultry meat in China’s market mainly depends on the centralized distribution of producing regions. This may be the main reason for the high prevalence levels of *Salmonella* in retail poultry meat across several regions of China. In addition, some potential reasons may be related to the differences in the retail environments [36], economic conditions [37], and market supervision [38] between these regions.

In the current study, *Salmonella* prevalence on chilled poultry meat was significantly higher than that on the poultry meat held at both ambient and frozen temperatures. The results showed that preservation methods of poultry meat may be a potential factor indicating cross-contamination at the retail level in China. Chilling is the most commonly utilized processing intervention to control *Salmonella* growth in the poultry meat production chain. Chilled poultry meat is usually kept at a low temperature by maintaining a monitored chill chain through portioning, packaging, transport, and retail storage [39,40]. In China, immersion chilling is employed more frequently. However, once a sample is contaminated with *Salmonella* during the immersion process, the contamination may spread among the whole batch of carcasses, leading to an increase in the prevalence of pathogens on finished products [41]. Consumers generally believe that freshly slaughtered meat has the advantages of higher nutritional value and superior taste [42]. Therefore, Chinese consumers have a preference for ambient meat (60% market share) over chilled meat (25% market share) or frozen meat (15% market share) [42]. Compared with the chilled poultry meat, fresh poultry meat purchased on the market can often be slaughtered, stripped, and eviscerated within 20 min [43] and may be less likely to experience cross-contamination. However, prevalence estimates are not sufficient to assess the probability and severity of illness to which people may be exposed. In a QMRA, implementation of quantitative exposure assessment depends on the concentration data of pathogens in food samples [44,45]. There is a general lack of quantitative data pertaining to *Salmonella* loads in food because most surveillance studies focus on the detection on a presence/absence basis. Therefore, viable cell numbers are often not known because most culture-based standard methods involve enrichment, while molecular methods (aside from RT-qPCR) do not assess viability. According to a few quantitative data on *Salmonella* in poultry meat, the average concentrations of *Salmonella* in the ambient stored samples are higher than that in the chilled samples [46,47]. Therefore, we speculate that although the pooled prevalence of *Salmonella* in freshly slaughtered poultry meat is low, its concentration levels are high, which may pose a greater risk to consumers.

The serotyping results of *Salmonella* isolates obtained from poultry meat in the current study revealed that *S*. Enteritidis, *S*. Indiana, and *S*. Typhimurium were the predominant serovars in poultry meat. The results of previous studies focusing on only one or several cities are consistent with the current nationwide data, indicating that *S*. Enteritidis, *S*. Indiana, and *S*. Typhimurium may be the main serotypes in poultry meat throughout China [6,46,48]. A global epidemiological meta-analysis of *Salmonella* serovars in animal-based foods indicated that *S*. Enteritidis was the most prevalent in Asia, Latin America, Europe, and Africa, while *S*. Typhimurium presented a global distribution [49]. There have been reports of *S*. Indiana in retail raw poultry meat in China since 2009, and this serotype appeared relatively late [50]. In particular, *S*. Enteritidis is most commonly associated with chickens and eggs and has a much smaller relationship with other food animal species [51]. What is more, *Salmonella* serovars Enteritidis, Typhimurium, and Indiana are also reported as the most common serotypes associated with human infections and outbreaks [52,53]. Thus, the high prevalence of these *Salmonella* serotypes in poultry meat indicates a significant risk to consumers. The dominant serotypes of *Salmonella* in food will change over time [54], which reminds us that the monitoring of the emergence and prevalence of different serotypes of *Salmonella* is essential for the better control of salmonellosis.

Nowadays, antimicrobial resistance is becoming an urgent threat and challenge to humans and the public. In the current study, more than half of *Salmonella* isolates were antimicrobial resistant. *Salmonella* isolates recovered from retail poultry meat showed a high frequency of resistance to nalidixic acid, tetracycline, ampicillin, chloramphenicol, trimethoprim/sulfamethoxazole, gentamicin, streptomycin, ciprofloxacin, sulfisoxazole, and ampicillin/sulbactam. Among them, whether in poultry meat or chicken, the highest rates of antimicrobial resistance were observed for nalidixic acid. Nalidixic acid is one of the most widely used antibacterial agents in feed additives and veterinary drugs worldwide. The uncontrolled use of quinolone in China will cause the emergence and increasing prevalence of antimicrobial-resistant *Salmonella*, complicating the treatment of *Salmonella* infections in humans and animals [10,55]. Resistance to tetracycline was the second most frequently observed, with tetracycline and ciprofloxacin also being front-line antibiotics for the treatment of salmonellosis [6]. However, *Salmonella* isolates in the current study were relatively susceptible to ciprofloxacin, a finding that is similar to a previous study in Iran [56]. Unfortunately, in several studies, *Salmonella* strains isolated from food, animals, and humans have been found to show multidrug-resistant (MDR) properties [57,58]. Furthermore, *S*. Indiana isolates with a high detection rate had been found to have high MDR levels [50]. The existence of MDR *Salmonella* isolates poses a major risk to public health, and food safety risk managers should continue to monitor their significant increase in resistance and implement further legislation on the prudent use of antimicrobials.

## 5. Conclusions

This study systematically reviewed the prevalence and epidemiology of *Salmonella* in retail raw poultry meat in China before 2020. *Salmonella* was more prevalent among chicken samples, especially chilled ones. Among the Chinese provincial regions, Shaanxi, Henan, Sichuan, and Beijing were high-risk areas for *Salmonella* contamination in poultry meat. The recovered *Salmonella* isolates belonged to multiple serovars. *S*. Enteritidis was the most commonly identified serovar in retail raw poultry meat in China. Meanwhile, poultry-derived *Salmonella* isolates showed a high prevalence of antimicrobial resistance, which represents a threat to human health. However, the qualitative sampling data of *Salmonella* accounts for the majority in the published reports on retail raw poultry meat across China. The scarcity of quantitative data on the contamination levels of *Salmonella* on poultry meat indicated the importance of future studies focusing on this topic and making possible quantitative microbial risk assessment studies.

The sampling conditions and laboratory methods of primary studies varied, limiting direct comparability between analyses. High levels of heterogeneity were found for the pooled prevalence of *Salmonella* for most sub-categories. It is concluded that future work should pay more attention to the synchronization of nationwide data and the collection of systematic sub-categories data. The baseline information on the prevalence, concentrations, serotypes, and antimicrobial resistance of *Salmonella* in various meat products from all provincial regions can be used not only to determine the severity of microbial contamination but also to serve as a point of reference for monitoring changes that occur over time. These data will be of great use in the development of effective risk management strategies in the future.

## Figures and Tables

**Table 1:** Meta-analysis results for mean prevalence of *Salmonella* in poultry by meat type based on the included reports.

Meat Category	Total	Positive	Pooled Prevalence (95% CI) ^a^	*τ* ^2 b^	*I* ^2 c^
Raw poultry overall (random effects)	21,824	5837	23.0% (19.8–26.6%)	0.8953	97.0%
Chicken	15,246	4716	26.4% (22.4–30.8%)	0.8821	96.9%
Duck	794	83	10.1% (5.3–18.2%)	0.7475	87.9%
Pigeon	292	66	22.6% (18.2–27.8%)	0.0000	0.0%
Other	5492	972	15.4% (12.0–19.5%)	0.2419	93.1%

^a^ 95% CI: 95% confidence interval; ^b^ *τ*^2^: between-study variance; ^c^ *I*^2^: inverse variance index.

**Table 2:** Meta-analysis results for mean prevalence of *Salmonella* in poultry by preservation type based on the included reports.

Preservation Type	Total	Positive	Pooled Prevalence (95% CI) ^a^	*τ* ^2^ ^b^	*I* ^2^ ^c^
Raw poultry overall (random-effects)	21,824	5837	23.0% (19.8%–26.6%)	0.8953	97.0%
Ambient	2825	649	17.2% (6.6%–37.8%)	2.3137	99.1%
Chilled	2066	974	42.1% (33.7%–51.0%)	0.2596	92.1%
Frozen	2173	535	25.3% (17.3%–35.4%)	0.6518	94.8%
Unknown	14,760	3679	21.3% (17.9%–25.1%)	0.7795	96.3%

^a^ 95% CI: 95% confidence interval; ^b^ *τ*^2^: between-study variance; ^c^ *I*^2^: inverse variance index.

## Data Availability

All data related to the research are presented in the article.
